# A 35-Year-Old Female With a Lupus Flare Presenting as Cardiac Tamponade: A Case Report

**DOI:** 10.7759/cureus.39050

**Published:** 2023-05-15

**Authors:** Tanushree Bhatt, Patrik Schmidt, Abeer Qasim, Priscilla Lajara, Aditya Ganti, Misbahuddin Khaja

**Affiliations:** 1 Internal Medicine, BronxCare Health System, Bronx, USA

**Keywords:** systemic lupus erythematosus, sle, cvd & sle, sle flare, large pericardial effusion, cardiac tamponade

## Abstract

Systemic lupus erythematosus (SLE) is a chronic autoimmune disorder that affects multiple organ systems, significantly impacting the cardiovascular system. One potential complication of acute SLE flare is the development of pericardial effusion which can lead to potentially life-threatening consequences if not promptly identified. In this report, we describe the case of a 35-year-old female with a known history of SLE who developed rapid-onset, large-volume pericardial effusion with tamponade during a lupus flare. She received emergency treatment involving pericardiocentesis and was administered high doses of glucocorticoid and immunosuppressive medication. As a result, the pericardial effusion gradually resolved, and the patient’s symptoms improved. This case emphasizes the significance of immediately identifying and managing swiftly progressing pericardial effusion in SLE patients. This is crucial as it can lead to severe and potentially lethal complications.

## Introduction

Systemic lupus erythematosus (SLE) is a chronic autoimmune disease characterized by the formation of immune complexes and antibodies that can affect various organs in the body, including the heart [1]. About >50% of lupus patients have some grade of pericardial involvement, but the majority remain asymptomatic [2]. Cardiac involvement in SLE usually manifests as pericarditis, myocarditis, endocarditis, valvular heart disease, or arrhythmias [3]. A rare but life-threatening cardiac complication of SLE is cardiac tamponade with an incidence of 1-3%, which occurs when pericardial fluid accumulates and compresses the heart [4]. Inflammation of the pericardium, acute renal failure, and anticoagulant therapy are some of the risk factors associated with the progression of tamponade in patients with SLE [5]. However, it is most common during acute flares of SLE when the immune system triggers a cascade of reactions to attack various organs, including the heart [6]. The condition presents with symptoms such as chest pain, shortness of breath, low blood pressure, and rapid heartbeat. If not urgently treated, it can result in cardiogenic shock and ultimately death [7].

Timely recognition and management of cardiac tamponade are imperative to ensure a favorable prognosis. Thus, it is vital to promote awareness of this infrequent yet severe complication of SLE among medical professionals and maintain a high level of vigilance for its signs and symptoms, especially during acute flares of the disease. The primary objective of this case study is to elaborate on the presentation, diagnosis, and management of a patient with cardiac tamponade in the context of an acute flare of SLE.

## Case presentation

A 35-year-old woman presented to the emergency room with multiple episodes of witnessed seizures. Preceding this, the patient had been experiencing cough, vomiting, and diarrhea for two days. She had a history of SLE with stage four lupus nephritis, Grave’s disease, hypertension, leukocytoclastic vasculitis, and anemia. She was recently hospitalized at another medical center for a lupus flare. Her medications included nifedipine, lisinopril, atenolol, methimazole, mycophenolate mofetil, hydroxychloroquine, voclosporin, and belimumab.

Upon arrival, her blood pressure was 207/135 mmHg. Additionally, she had a rapid heartbeat of 114 beats per minute, was tachypneic, hypoxic, and had a fever. A physical examination revealed diminished breath sounds bilaterally with distant heart sounds. She was intubated for respiratory failure in the emergency room. Her laboratory findings were significant for anemia, leucocytosis, low C3 and C4, elevated erythrocyte sedimentation rate, and C-reactive protein, as shown in Table 1.

**Table 1:** Laboratory findings on admission.

Laboratory parameters	Results	Reference range and units
Sodium	139 mEq/L	135–145 mEq/L
Potassium	2.9 mEq/L	3.5–5.0 mEq/L
Blood urea nitrogen	19 mg/dL	6–20 mg/dL
Creatinine	1.3 mg/dL	0.5–1.5 mg/dL
Hemoglobin	8.5 g/dL	12–16 g/dL
White blood cell	24.1 K/µL	4.8–10.8 K/µL
Platelet	196 K/µL	150–400 K/µL
C-reactive protein	63.38 mg/L	<5 mg/L
Erythrocyte sedimentation rate	57 mm/hour	<20 mm/hour
Anti-nuclear antibody	1:320	<1:40
Anti-deoxyribonucleic acid antibody	133 IU/mL	<4 IU/mL
C3	<40 mg/dL	90–150 mg/dL
C4	<6 mg/dL	16–47 mg/dL
Thyroid-stimulating hormone	0.01 mIU/L	0.4–4.5 mIU/L
T3	212	60–181 ng/dL
Free thyroxine	2.98 ng/dL	0.8–2 ng/dL

**Figure 1:**
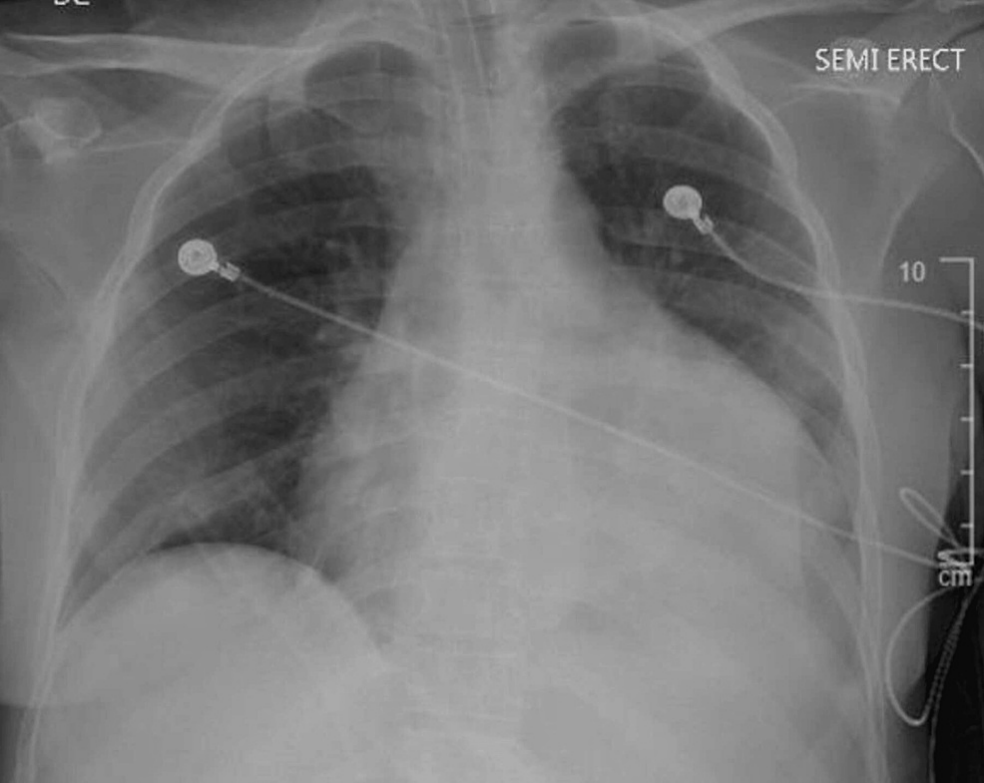
Chest X-ray on admission showing left perihilar and lower lobe infiltrates concerning pneumonia and cardiomegaly.

Her urinalysis showed the presence of red blood cells (>30 cells/high-power field) and protein (600 mg/dL). She had an abnormal thyroid panel suggestive of thyrotoxicosis (Table 1). A computed tomography scan of the head showed vasogenic edema in the occipital and parietal regions. A chest X-ray showed left perihilar and lower lobe infiltrates concerning pneumonia and cardiomegaly, as demonstrated in Figure 1.

**Figure 2:**
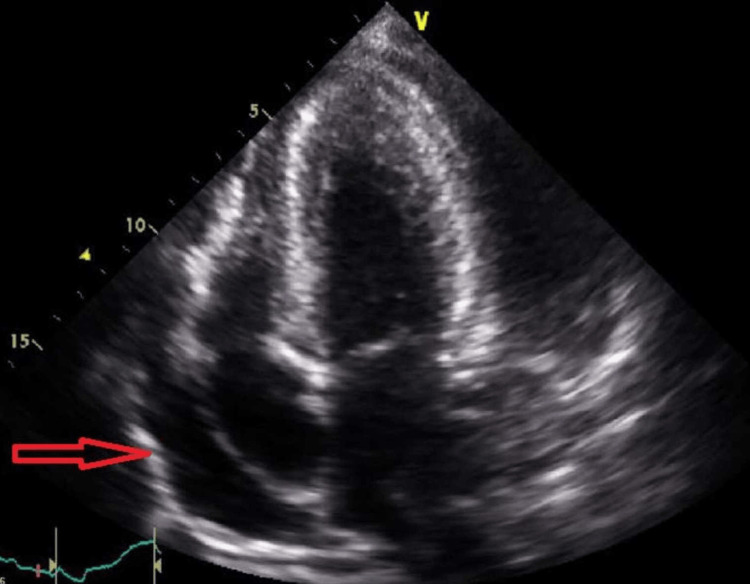
Echocardiography demonstrating (red arrow) a large pericardial effusion present on initial imaging.

Her sputum culture grew *Hemophilus influenzae*. Treatment was initiated with lorazepam, levetiracetam, and broad-spectrum antibiotics (vancomycin and meropenem). The patient underwent a lumbar puncture and meningitis was ruled out (white blood cell count 0, glucose 55 mg/dL, protein 26 mg/dL, negative gram stain and culture). Echocardiography showed cardiac tamponade with an ejection fraction of 41% (Figure 2).

**Figure 3:**
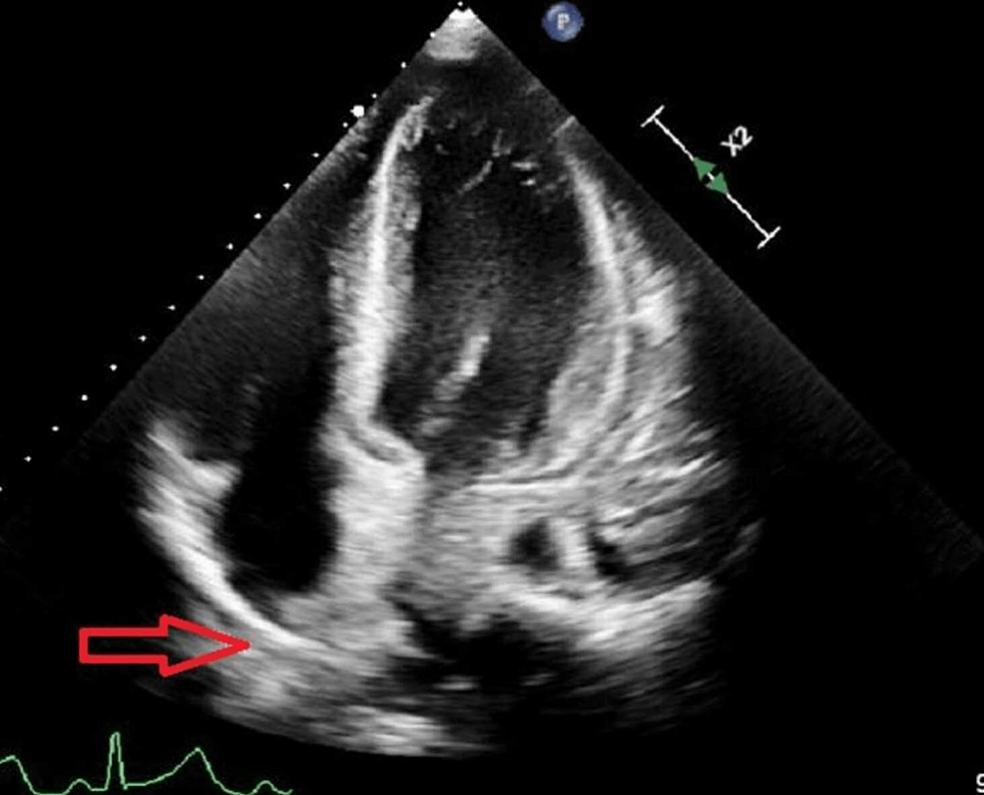
Repeat echocardiography after pericardiocentesis showing resolution of previously observed pericardial effusion. The red arrow indicates the region previously occupied by the pericardial effusion (now no longer present).

**Figure 4:**
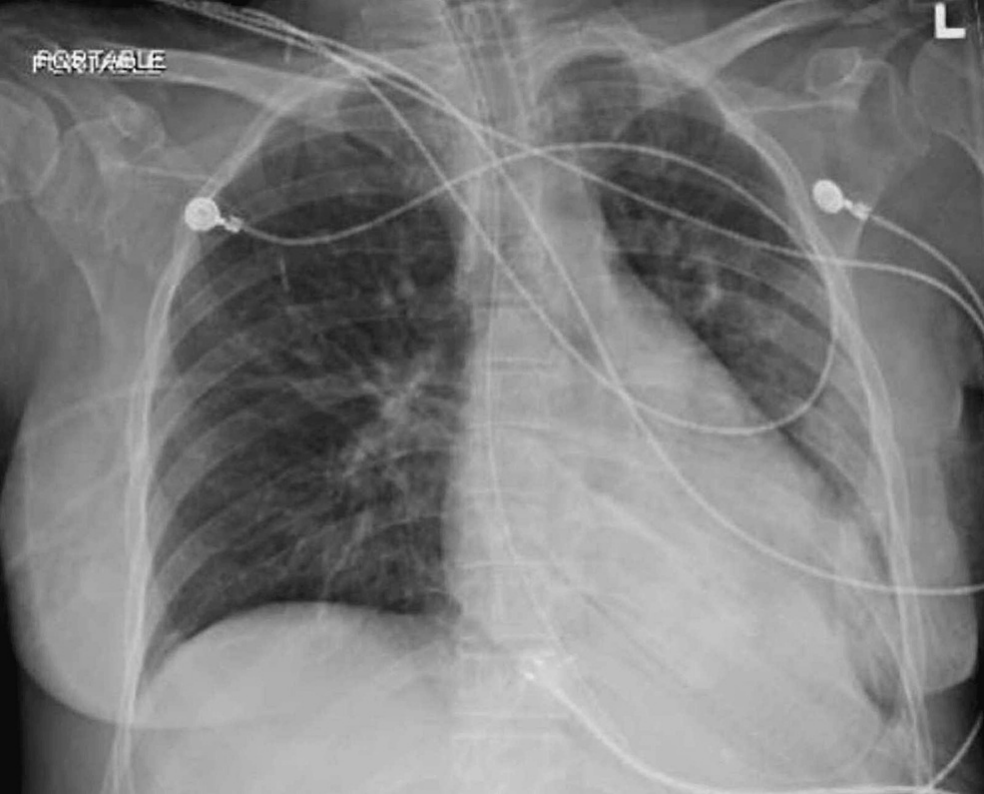
Chest X-ray after pericardiocentesis showing significant reduction in cardiac silhouette after drainage of pericardial fluid.

The patient underwent urgent pericardiocentesis through a sub-xiphoid approach and 750 mL of serous fluid was drained. Repeat echocardiography (Figure 3) and chest X-ray (Figure 4) showed a resolution of pericardial effusion with an improvement of ejection fraction to 78%.

Rheumatology was consulted and the patient was deemed to be in an active lupus flare due to the presence of fever, cardiac tamponade, and low complement level. Treatment was started with a pulse dose of steroids, followed by a tapering course, hydroxychloroquine, and cyclophosphamide. The patient’s course was complicated by acute renal failure causing anuria, likely due to the lupus flare, for which she was started on temporary hemodialysis. Her hospital course was further complicated by lung collapse requiring emergent fiberoptic bronchoscopy with bronchoalveolar lavage and a repeat pericardial effusion requiring placement of a pericardial drain. She was eventually weaned off the ventilator after improvement in her respiratory function. She continued to receive hydroxychloroquine daily, along with cyclophosphamide infusions every two weeks, and a tapering course of prednisone. Subsequent echocardiography and chest X-ray confirmed slow but gradual improvement of her pericardial effusion, as shown in Figures 2-4, which was followed up by the removal of her pericardial drain and discharge with close cardiac and rheumatological outpatient follow-up.

## Discussion

SLE is an autoimmune connective tissue disorder that is characterized by the formation of immune complexes and autoantibodies that affect multiple organ systems. A flare of SLE refers to worsening disease activity in one or more organ systems, as evidenced by clinical findings and laboratory markers [8]. The cardiovascular complications related to SLE include myocarditis, pericarditis, conduction abnormalities, pericardial effusion, and cardiac tamponade. Cardiac tamponade is one of the rare complications of SLE, occurring in about 1% of SLE patients, which can have fatal consequences if left untreated [1].

The prevalence of SLE is eight to ten times higher in women than in men [4]. A retrospective study conducted with 409 patients showed that 5.9% of patients with SLE developed cardiac tamponade [5]. Cardiac tamponade is the sequelae of pericardial effusion that causes excessive accumulation of fluid in the pericardial space, restricting the filling of the cardiac chambers. This results in decreased right ventricular filling and left ventricular cardiac output, leading to hypotension and possibly cardiac arrest [6]. Common findings in cardiac tamponade include acute-onset chest pain, dyspnea, tachypnea, tachycardia, venous congestion, syncope, presyncope, and hypotension. Classic physical examination findings of cardiac tamponade include Beck’s triad (jugular venous distention, hypotension, and muffled heart sounds) and pulsus paradoxus. While there are certain electrocardiogram findings that are associated with cardiac tamponade, such as low-voltage QRS and electrical alternans, a study done by Chandra et al. suggested that these cardiac findings are neither sensitive nor specific, with electrical alternans being present in only 23.7% of patients and low-voltage QRS present in 29% of patients [7]. Echocardiogram studies can also be helpful as pericardial fluid collections exceeding 75-100 mL can be visualized on echocardiogram, along with atrial and ventricular collapse/compression, swinging of the heart in the pericardial sac, and dilated inferior vena cava [9]. Recent studies have shown an association between laboratory studies such as low complement levels with an increased risk of developing cardiac tamponade [5]. Treatment involves immediate removal of pericardial fluid, either by pericardiocentesis or pericardiotomy, and initiation of high-dose corticosteroids and intravenous cyclophosphamide, as these have shown to be effective in alleviating symptoms and preventing recurrent fluid accumulation in SLE-induced cardiac tamponade [10].

Our patient is a 35-year-old female who presented to the emergency due to hypertensive emergency, seizures, and syncope, underwent an echocardiogram, and was found to have cardiac tamponade in the setting of the pericardial effusion, requiring emergent pericardiocentesis. Cardiac tamponade is diagnosed based on clinical presentation; however, electrocardiogram, echocardiogram, and chest X-ray can be useful in diagnosis. Electrocardiogram findings are typically electrical alternans and low voltage. Prompt diagnosis is imperative to reduce the risk of mortality and improve long-term patient outcomes during acute disease flares.

## Conclusions

Pericardial tamponade is a serious complication of an acute SLE flare. During the acute inflammatory changes following an SLE flare, rapid fluid shifts lead to the accumulation of pericardial effusion that causes a dynamic restriction in cardiac function and precipitation of acute heart failure. Our case highlights this rapid deterioration in a young patient with acute hemodynamic instability secondary to a large pericardial effusion that required emergency pericardiocentesis to quickly reverse her cardiac dysfunction. She was subsequently treated with high-dose cyclophosphamide and hydroxychloroquine along with a prednisone taper which led to her clinical improvement both in terms of her systemic inflammation and degree of pericardial effusion. Prompt investigation for pericardial effusions in the setting of SLE flare is an essential consideration in patients with hemodynamic compromise, as its identification and rapid management can result in improved patient outcomes.
